# Tubulointerstitial De Novo Expression of the α8 Integrin Chain in a Rodent Model of Renal Fibrosis – A Potential Target for Anti-Fibrotic Therapy?

**DOI:** 10.1371/journal.pone.0048362

**Published:** 2012-11-08

**Authors:** Andrea Hartner, Carlos Menendez-Castro, Nada Cordasic, Ines Marek, Gudrun Volkert, Bernd Klanke, Wolfgang Rascher, Karl F. Hilgers

**Affiliations:** 1 Department of Pediatrics and Adolescent Medicine, University Hospital of Erlangen, Erlangen, Germany; 2 Department of Nephrology and Hypertension, University Hospital of Erlangen, Erlangen, Germany; Baker IDI Heart and Diabetes Institute, Australia

## Abstract

In the normal kidney, the α8 integrin chain is expressed only on mesangial cells and vascular smooth muscle cells. α8 integrin ligates several matrix molecules including fibronectin, osteopontin and fibrillin-1. Recently, we detected de novo expression of α8 integrin on epithelial cells in renal cysts. We hypothesized that the α8 integrin chain is induced in tubular epithelia undergoing dedifferentiation and contributes to the fibrotic response in the tubulointerstitium (TI) after unilateral ureteral obstruction (UUO). After induction of UUO in rats by ligation of the right ureter, increased expression of the α8 integrin chain and its ligands was observed. In the TI, α8 integrin was localized to cytokeratin-positive epithelial cells and to interstitial fibroblasts; and colocalized with its ligands. In mice underexpressing α8 integrin UUO led to collagen deposition and fibroblast activation comparable to wild types. Mice lacking α8 integrin showed even more TI damage, fibroblast activation and collagen deposition after UUO compared to wild type mice. We conclude that the expression of the α8 integrin chain and its ligands is strongly induced in the TI after UUO, but underexpression of α8 integrin does not attenuate TI fibrosis. Mice lacking the α8 integrin chain are even more susceptible to TI damage than wild type mice. Thus, interactions of α8 integrin with its ligands do not seem to contribute to the development or progression of TI fibrosis in UUO. Targeting α8 integrin might not be a useful approach for anti-fibrotic therapy.

## Introduction

Integrins, serving as receptors for extracellular matrix molecules, contribute to the maintenance of the structural and functional integrity of tissues and organs [1]. On the other hand, a dysregulated expression of integrins can also add to pathologic changes like tissue fibrosis: In a rat model of crescentic glomerulonephritis α1β1 integrin is overexpressed in glomeruli and the tubulointerstitium and treatment with an inhibitory antibody to α1β1 integrin reduced renal scarring and ameliorated renal survival [2]. Similarly, an inhibition of αvβ3 using specific RGD peptides resulted in a reduction of glomerulosclerosis during experimental Habu glomerulonephritis [3]. In Alport mice, the expression of αvβ6 integrin correlates with renal fibrosis and blocking of this integrin results in reduced deposition of collagen matrix [4]. The expression of the α5 integrin chain was induced in tubular epithelial cells after unilateral ureteral obstruction and facilitated epithelial to mesenchymal transition in these cells [5]. Thus, integrin signaling pathways seem to contribute to fibrotic changes in different renal diseases. For this reason integrins appear to be promising targets for more specific therapeutic approaches to prevent renal fibrosis [6]. On the other hand, integrins also serve to maintain tissue integrity. A complete lack of α1β1 integrin even leads to more severe glomerulosclerosis after experimental glomerular injury [7].

The α8 integrin chain dimerizes solely with the β1 chain to form α8β1 integrin, which is a receptor for fibronectin, vitronectin, tenascin C, osteopontin, nephronectin and fibrillin-1 [8]–[12]. In the normal kidney, the α8 integrin chain is expressed exclusively in vascular smooth muscle cells and mesangial cells [13]. The expression of α8 integrin is induced in various rat models of tissue fibrosis: in the scarring liver, lung and heart [14], [15]. Own studies revealed an overexpression of α8 integrin in glomerulosclerosis [13]. Recently de novo expression of the α8 integrin chain in epithelial cells in the wall of renal cysts was observed [16]. We speculate that this de novo expression may be a more widespread feature of renal epithelial cells undergoing dedifferentiation. We therefore tested the hypothesis that the α8 integrin chain is expressed in dedifferentiating tubular epithelial cells during tubulointerstitial injury and that it contributes to the fibrotic response of the kidney. As a model of tubulointerstitial fibrotic injury, we performed unilateral ureteral obstruction (UUO) and studied α8 integrin expression patterns in this context. To investigate the functional role of α8 integrin for renal fibrosis, we induced UUO in mice with a deficiency for the α8 integrin chain.

## Materials and Methods

### Animal procedures

Animals were housed in a room maintained at 22±2°C, exposed to a 12 hour dark/light cycle with free access to standard chow (#V1534, Sniff, Soest, Germany) and tap water. All procedures performed on animals were done in accordance with the NIH Guide for the Care and Use of Laboratory Animals and were approved by the local government authorities (Regierung von Mittelfranken, approval number AZ # 54-2532.1-17/08) after evaluation of the local government's review board for animal research ethics. All surgery was performed under isoflurane or sodium pentobarbital anesthesia, and all efforts were made to minimize suffering. If judged necessary by a veterinarian, buprenorphine hydrochloride was injected to prevent of relief suspected pain or discomfort.

Male Sprague-Dawley rats of 180–220 g body weight were purchased from Charles River (Sulzfeld, Germany). For histological evaluations, 15 rats underwent unilateral ureteral obstruction. The right ureter was ligated with 4.0 silk at two separate locations. Rats were sacrificed by exsanguination under deep anesthesia at day 2 (n = 5), day 7 (n = 5) and day 14 (n = 5) after unilateral ureteral obstruction. The left kidneys were used as controls. At day 2 after ligation fibrotic changes were not significantly different from controls, while at day 7 after ligation fibrotic changes were clearly observed. At day 14 after ligation fibrotic changes observed were not essentially different from the changes detected at day 7. Thus, we chose the day 7 time point for further investigations.

α8 integrin-deficient mice were a generous gift of Dr. Ulrich Muller (Scripps Institute, LaJolla, USA) and bred in our animal facilities. Male mice with a homozygous deletion of the α8 integrin (α8−/−) bearing two kidneys at an average weight of 20 g were used for experiments. Age and weight matched male heterozygous (α8+/−) and wild type (α8+/+) litters served as controls. For unilateral (right) ureteral obstruction 8 α8+/+, 8 α8+/− and 8 α8−/− were used. As controls, 8 α8+/+, 8 α8+/− and 8 α8−/− were sham operated. Mice were sacrificed at day 7 after unilateral ureteral obstruction.

One day before sacrifice, animals were kept in metabolic cages for 24 hours to collect urine. Blood samples were obtained at sacrifice. At the day of sacrifice, animals were equipped with a carotid artery catheter under general anesthesia and intraarterial blood pressure was measured in conscious animals 2 hours after anesthesia. Animals were exsanguinated in deep general anesthesia and the left ventricle and the kidneys were removed. Cortical tissue from each kidney was frozen in liquid nitrogen for RNA analyses. One part of the kidney was frozen in Cryoblock (medite Medizintechnik, Burgdorf, Germany) to prepare cryosections. Other parts of the kidney were fixed in methyl Carnoys solution or in paraformaldehyde for embedding in paraffin.

### Measurement of physiologic parameters

Urinary albumin excretion was measured using an ELISA (CellTrend, Luckenwalde, Germany). Serum and urinary creatinine and serum urea were assessed using an automated analyzer (Integra 800, Roche Diagnostics, Mannheim, Germany).

### Real time RT-PCR

Renal cortical tissue (10 mg) was homogenized in 500 µl of RLT buffer reagent (Qiagen, Hilden, Germany) with an ultraturrax for 30 seconds, and total RNA was extracted with RNeasy Mini columns (Qiagen) according to the standard protocol. First-strand cDNA was synthesized with TaqMan reverse transcription reagents (Applied Biosystems, Darmstadt, Germany) using random hexamers as primers. Final RNA concentration in the reaction mixture was adjusted to 0.1 ng/µL. Reactions without Multiscribe reverse transcriptase were used as negative controls for genomic DNA contamination. RT-products were diluted 1∶1 with dH_2_O before PCR procedure. PCR was performed with an ABI PRISM 7000 Sequence Detector System and SYBR Green reagents (Applied Biosystems) according to the manufacturer's instructions. Primers used in this study are listed in table 1. Primer pairs were designed using the Primer Express software (Perkin Elmer, Foster City, CA, USA) except for TGFβ [17], collagen I [18], vitronectin [19], nephronectin [20], fibronectin [21] and osteopontin [22]. The relative amount of the specific mRNA was normalized with respect to 18S rRNA. All samples were run in triplicates.

**Table 1:** Primer pairs for Sybr green and QT PCR analysis.

	forward	reverse
Rat primer:		
TGFβ	5′-TGG AAG TGG ATC CAC GCG CCC AAG G-3′	5′-GCA GGA GCG CAC GAT CAT GTT GGA C-3′
CTGF	5′-TGT GCA CTG CCA AAG ATG GT-3′	5′-GGT ACA CGG ACC CAC CGA-3′
LTBP-1	5′-CGG ATC CCC CTA TGA TCT CA-3′	5′-TGA CGA GGC GGT AGC AGG-3′
Collagen I	5′-TCA CCT ACA GCA CGC TTG-3′	5′-GGT CTG TTT CCA GGG TTG-3′
α8 integrin	5′-TCC AAA TCA GAA GCT CCA ACA A-3′	5′-CGC TCA CGA AAT TGC TGT CA-3′
β1 integrin	5′-AAG TCC CAA GTG CCA TGA GG-3′	5′-CTG CAG GCT CCA CAC TCA AAT-3′
Fibrillin-1	5′-TGC TCT GAA AGG ACC CAA TGT-3′	5′-CGG GAC AAC AGT ATG CGT TAT AAC-3′
Fibronectin	5′-TTG CAA CCC ACC GTG GAG TAT GTG-3′	5′-CTC GGT AGC CAG TGA GCT TAA CAC-3′
Osteopontin	5′-AAA GTG GCT GAG TTT GGC AG-3′	5′-AAG TGG CTA CAG CAT CTG AGT GT-3′
	probe 5′ (FAM)-TCA GAG GAG AAG GCG CAT TAC AGC A-(TAMRA)3′	
Vitronectin	5′-GCT GAC CAA GAG TCA TGC AA-3′	5′-GGT TTC CTC CGG GTA GTC AT-3′
Nephronectin	5′-AGC CAA CAA CAA GAC CTA CAC-3′	5′-GCC GTG GAA TGA ACA CAA TCT C-3′
18S	5′-TTG ATT AAG TCC CTG CCC TTT GT-3′	5′-CGA TCC GAG GGC CTC ACT A-3′
Mouse primer:		
α8 integrin	5′-AGA ATG ATT ACC CAG ATT TGC TTG T-3′	5′-GCT ACT TTC CCT TTT CCA AAT GC-3′
Smooth muscle actin	5′-CCC TGA AGA GCA TCC GAC AC-3′	5′-GCC TTA GGG TTC AGT GGT GC-3′
Collagen I	5′-TCA CCT ACA GCA CCC TTG TGG-3′	5′-CCC AAG TTC CGG TGT GAC TC-3′
Fibronectin	5′-TGT GAC CAG CAA CAC GGT G-3′	5′-ACA ACA GGA GAG TAG GGC GC-3′
Osteopontin	5′-TTT GCT TTT GCC TGT TTG GC-3′	5′-CAG TCA CTT TCA CCG GGA GG-3′
Fibrillin-1	5′-ACA GGT CAA TGC AAC GAT CG-3′	5′-GCA TAT GTT CGG GAT TTC TTG G-3′

### Immunohistochemical analyses

Staining of cryostat and paraffin sections was performed as previously described [23]. The primary rabbit antibody to α8 integrin (Ulrich Muller, Scripps Institute, LaJolla, USA) and the primary rabbit antibody to fibrillin-1 (Dieter Reinhardt, McGill University, Montreal, Canada) were used on cryopreserved tissue at a dilution 1∶500. CY3 labeled anti-rabbit IgG (DAKO Diagnostica, Hamburg, Germany) was used as secondary antibody. Double immunofluorescence for α8 integrin and the cell markers smooth muscle actin (DAKO) and cytokeratin (DAKO) as well as for the α8 integrin ligands fibronectin (Life Technologies, Darmstadt, Germany), osteopontin (Jack Kleinman, Medical College of Wisconsin, Milwaukee, USA) and fibrillin-1 was performed as described [23]. Detection and quantification of α-smooth muscle actin, vimentin (Progen, Heidelberg, Germany), collagen I (Biogenesis, Poole, England), fibronectin, osteopontin was carried out in paraffin embedded renal tissue as described [23].

Evaluation of the expansion of various cell markers and matrix molecules was done with a Leitz Aristoplan microscope (Leica Instruments) in 5 (mice) to 10 (rats) non-overlapping medium power views using Metavue software (Visitron Systems, Puchheim, Germany). The stained area was expressed as percentage of the total area of renal cross section.

### Renal histology

Tubulointerstitial injury was evaluated in kidney sections stained with period acid-Schiff's (PAS) reagent. Tubulointerstitial injury was assessed in 5 non-overlapping medium power randomly sampled views per kidney section using a tubulointerstitial damage score grading tubular atrophy, tubular dilatation, interstitial fibrosis and interstitial inflammation as follows: grade 0, no change; grade 1, lesions involving less than 25% of the area; grade 2, lesions affecting 25–50%; grade 3, lesions involving more than 50% and grade 4 involving (almost) the entire area.

### Analysis of data

A student's t-test was used to test significance of difference between two groups. Otherwise, analysis of variance (ANOVA), followed by posthoc Bonferroni test, was used to test significance of differences between groups. A p-value of less than 0.05 was considered significant. The procedures were carried out using the PASW Statistics 18 software (SPSS Inc., Chicago, USA). Values are displayed as means ± SEM.

## Results

### After UUO, α8 integrin is de novo expressed in tubular epithelial cells and fibroblasts and colocalizes with its ligands fibronectin, osteopontin and fibrillin-1

In response to UUO, an increase in the expression of the profibrotic cytokines connective tissue growth factor (CTGF) and TGFβ as well as of the TGFβ-associated matrix molecule latent TGFβ binding protein (LTBP-1) was observed (table 2). Concomitantly, the expression of collagen I was induced and immunoreactivity of α-smooth muscle actin and vimentin as markers of activated myofibroblasts was more abundant (table 2).

**Table 2:** Expression of profibrotic cytokines, matrix molecules and cell activation markers in the kidney after UUO.

	co	UUO	p
CTGF (fold induction)	1.00±0.17	3.13±0.75	* 0.037
TGFβ (fold induction)	1.00±0.29	123.75±32.43	* 0.004
LTBP-1 (fold induction)	1.00±0.32	11.31±0.81	* <0.001
collagen I (fold induction)	1.00±0.30	13.79±4.80	* 0.019
integrin chain β1 (fold induction)	1.00±0.16	2.44±0.33	* 0.004
fibronectin (fold induction)	1.00±0.25	7.50±1.11	* 0.001
osteopontin (fold induction)	1.00±0.15	45.87±14.06	* 0.018
fibrillin-1 (fold induction)	1.00±0.33	64.47±4.29	* <0.001
vitronectin (fold induction)	1.00±0.29	14.10±15.06	0.347
nephronectin (fold induction)	1.00±0.22	1.34±0.53	0.541
fibronectin (% area stained)	2.45±0.58	9.22±2.65	* 0.023
osteopontin (% area stained)	5.64±3.02	18.82±2.97	* <0.001
fibrillin-1 (% area stained)	5.68±0.41	36.80±2.45	* <0.001
α-sma (% area stained)	2.06±0.37	14.06±4.31	* 0.031
vimentin (% area stained)	1.83±0.16	15.84±2.07	* <0.001

Co, control kidney without ureteral ligation; UUO, kidney with ureteral ligation. α-sma, α-smooth muscle actin; data are means±sem.

**Figure 1:**
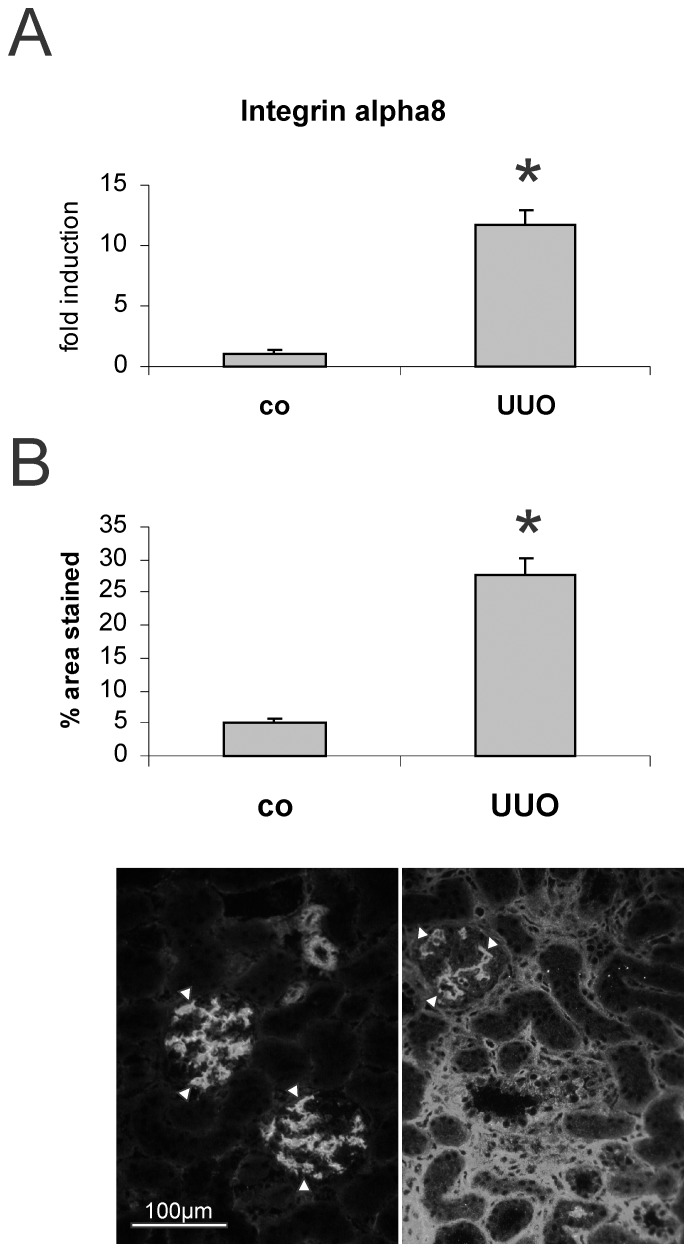
α8 integrin expression in the kidney after unilateral ureteral obstruction (UUO). A: mRNA expression in cortical renal tissue. B: immunohistochemical evaluation of α8 integrin in the kidney after UUO with exemplary photomicrographs. White arrowheads indicate physiologic mesangial and vascular α8 integrin expression. Co, contralateral kidney; * p<0.05.

**Figure 2:**
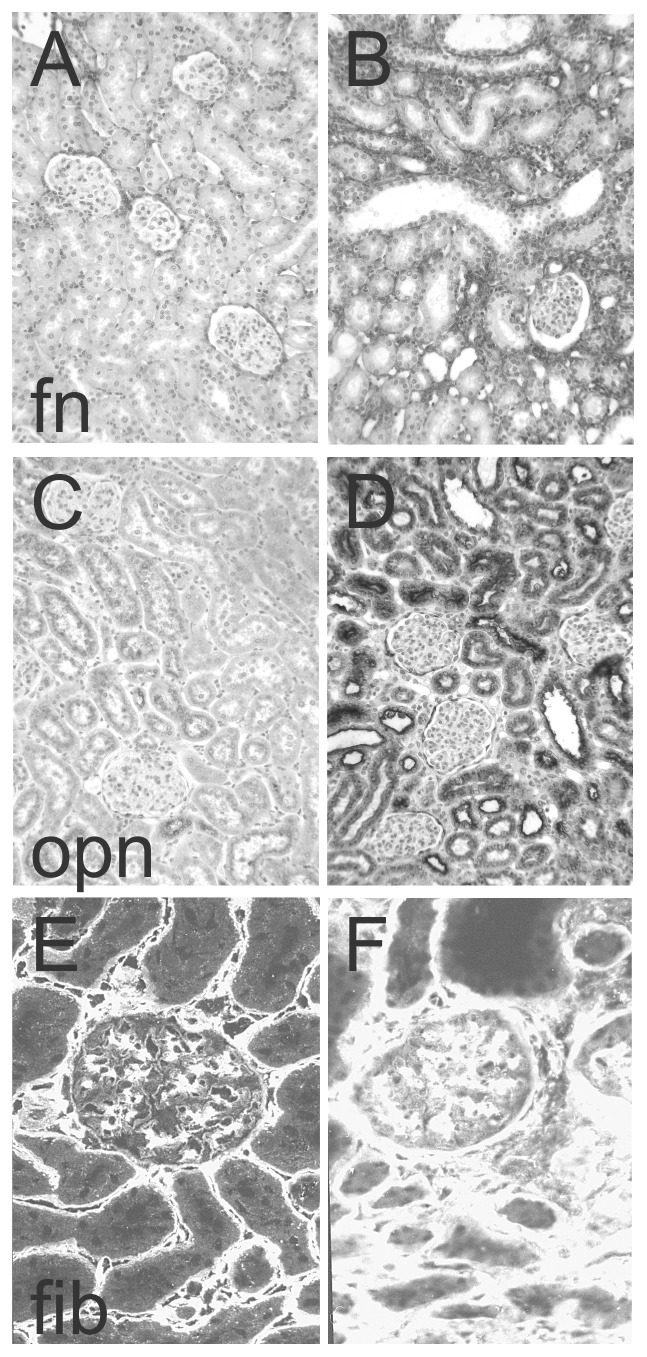
Exemplary photomicrographs of the immunohistochemical detection of A+B fibronectin (fn), C+D osteopontin (opn) and immunofluorescent detection of E+F fibrillin-1 (fib) in control kidneys (A, C, E) and in kidneys after unilateral ureteral obstruction (B, D, F). Please note that A, B, C and D are enzymatic stainings which result in a dark stain, while E and F are fluorescent stainings resulting in a bright stain.

The mRNA expression and immunoreactivity of α8 integrin was significantly increased after UUO (figure 1). While confined to mesangial cells and vascular smooth muscle cells in the control kidney (white arrowheads), tubulointerstitial staining for α8 integrin was observed after UUO (figure 1B). The expression of the α8 integrin chain dimerization partner β1 integrin chain was also induced (table 2). As binding of integrins to their ligands is essential for integrin signaling, we also investigated the expression of the ligands of α8 integrin: The expression of vitronectin and nephronectin was low (at detection limits) and was not increased after UUO (table 2). In contrast, the expression and immunoreactivity for fibronectin, osteopontin and fibrillin-1 was significantly induced in kidneys of UUO animals (table 2). Fibronectin and fibrillin-1 were detected in the tubulointerstitium (figure 2), while osteopontin was detected in tubular epithelial cells (figure 2).

**Figure 3:**
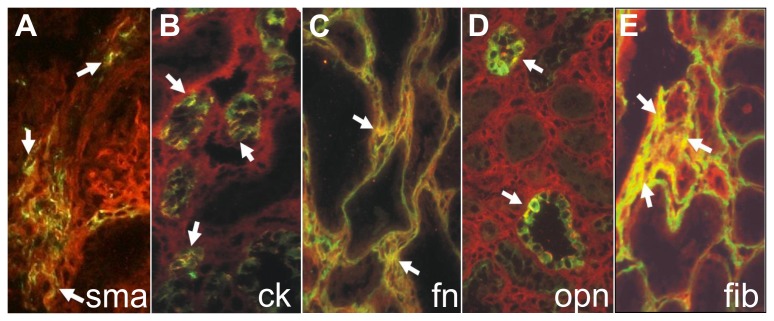
Colocalization of α8 integrin with cell markers and ligands. Double immunofluorescent analyses detecting α8 integrin (red) and A: α-smooth muscle actin (sma, green), B: cytokeratin (ck, green), C: fibronectin (fn, green), D: osteopontin (opn, green), E: fibrillin-1 (fib, green). Colocalization presents as yellow fluorescence (white arrows).

To determine the cell types expressing the α8 integrin chain, double immunofluorescent studies were performed for α8 integrin and the cell markers cytokeratin for epithelial cells and α-smooth muscle actin for activated interstitial fibroblasts. There was a partial colocalization observed with both markers (figure 3A and B) arguing for a de novo expression of α8 integrin in tubular epithelial cells and interstitial fibroblasts in this model in addition to the established expression of α8 integrin in mesangial cells and vascular smooth muscle cells. Moreover, colocalization of α8 integrin with its ligands fibronectin and fibrillin-1 was observed in the tubular interstitium (figure 3C and E). Colocalization of α8 integrin with osteopontin was observed in tubular epithelial cells (figure 3D).

### The expression of α8 integrin is reduced in heterozygous α8 integrin-deficient (α8+/−) mice and is barely detectable in homozygous α8 integrin-deficient (α8−/−) mice after UUO

To assess the contribution of de novo expressed α8 integrin to tubulointerstitial fibrosis, UUO was induced in α8 integrin-deficient mice. As our studies in rats had revealed overt fibrotic changes in the obstructed kidney after 7 days of UUO, this time point was chosen for our further experiments. The expression of α8 integrin was compared in the genotypes after UUO. Kidneys of α8+/− mice expressed reduced amounts of α8 integrin (0.48±0.08 fold induction versus 1.00±0.14 in wild types, p<0.05), while expression levels of α8 integrin in α8−/− mice was at detection limits (0.0012±0.0003 fold induction). Expression of the α8 integrin ligands fibronectin, osteopontin and fibrillin-1 was increased after UUO, without any significant differences between wild types and α8+/− mice or α8−/− mice (table 3).

**Table 3:** Expression of α8 integrin ligands, α-smooth muscle actin (α-sma) and collagen I in the kidney of α8 Integrin underexpressing mice after UUO.

	WT	α8+/−	α8−/−
fibronectin (fold induction)	17.1.82±0.16*	19.45±2.47*	16.69±2.17*
osteopontin (fold induction)	26.14±2.58*	27.25±4.80*	32.08±5.84*
fibrillin-1 (fold induction)	8.46±0.82*	11.63±1.33*	10.14±1.42*
α-sma (fold induction)	2.50±0.409*	3.37±0.44*	4.01±0.42* #
collagen I (fold induction)	30.71±4.91*	33.89±4.16*	35.46±4.49*

UUO, unilateral ureteral obstruction; WT, wild type; α8+/−, mice with a heterozygous deficiency for α8 integrin; α8−/−, mice with a homozygous deficiency for α8 integrin. Values are fold induction of the sham WT control group. Data are means±sem.

* p<0.05 UUO versus sham,

# p<0.05 α8−/− versus wild type UUO.

### Renal fibrosis after UUO is more severe in α8−/− mice but unchanged in α8+/− mice compared to wild type mice

After 7 days of UUO, body weights, left ventricular weights and blood pressure were comparable between all experimental groups (table 4). Relative kidney weights tended to be higher after UUO, reaching statistical significance only in α8−/− mice (table 4). Albumin excretion was not significantly different between groups (table 4). Creatinine clearance tended to be reduced in all genotypes after UUO, while serum creatinine values tended to be increased after UUO in wild type and α8−/− mice and increased significantly in α8+/− mice (table 4). In α8−/− mice, serum urea increased in response to UUO (table 4) and UUO led to higher serum urea levels in α8−/− mice compared to wild types (table 4).

**Table 4:** Body and organ weights and physiological parameters of wild type and α8 integrin-deficient mice after UUO.

	WT sham	WT UUO	α8+/− sham	α8+/− UUO	α8−/− sham	α8−/− UUO
Body weight (g)	24.27±0.72	25.29±0.55	25.57±0.46	23.58±0.66	23.56±0.69	22.58±57
Rel. left kidney weight (% g/g)	0.552±0.031	0.628±0.022	0.558±0.0150	0.621±0.024	0.433±0.017	0.549±0.027* #
Rel. right kidney weight (% g/g)	0.590±0.010	0.607±0.035	0.594±0.026	0.646±0.038	0.425±0.021	0.522±0.029
Rel. left ventricular weight (% g/g)	0.323±0.006	0.346±0.010	0.328±0.006	0.356±0.021	0.330±0.008	0.346±0.007
Mean arterial pressure (mm Hg)	122.9±3.0	133.6±3.4	123.3±4.69	133.7±6.43	121.6±4.0	123.7±2.5
Albuminuria (µg/d)	0.042±0.009	0.039±0.009	0.038±0.007	0.028±0.005	0.070±0.010	0.053±0.007
Creatinine clearance (ml/min)	0.374±0.103	0.217±0.047	0.246±0.048	0.150±0.023	0.264±0.033	0.179±0.028
Serum creatinine (mg/dl)	0.066±0.007	0.101±0.011	0.076±0.007	0.157±0.039*	0.088±0.007	0.136±0.0143
Serum urea (mg/dl)	60.08±2.50	69.84±6.32	59.06±2.16	74.99±3.20	67.57±5.11	91.39±6.35* ^, ^ #

WT, wild type; UUO, unilateral ureteral obstruction, α8+/−, mice with a heterozygous deficiency for α8 integrin; α8−/−, mice with a homozygous deficiency for α8 integrin. Data are means±sem.

* p<0.05 UUO versus sham,

# p<0.05 α8−/− versus wild type UUO.

**Figure 4:**
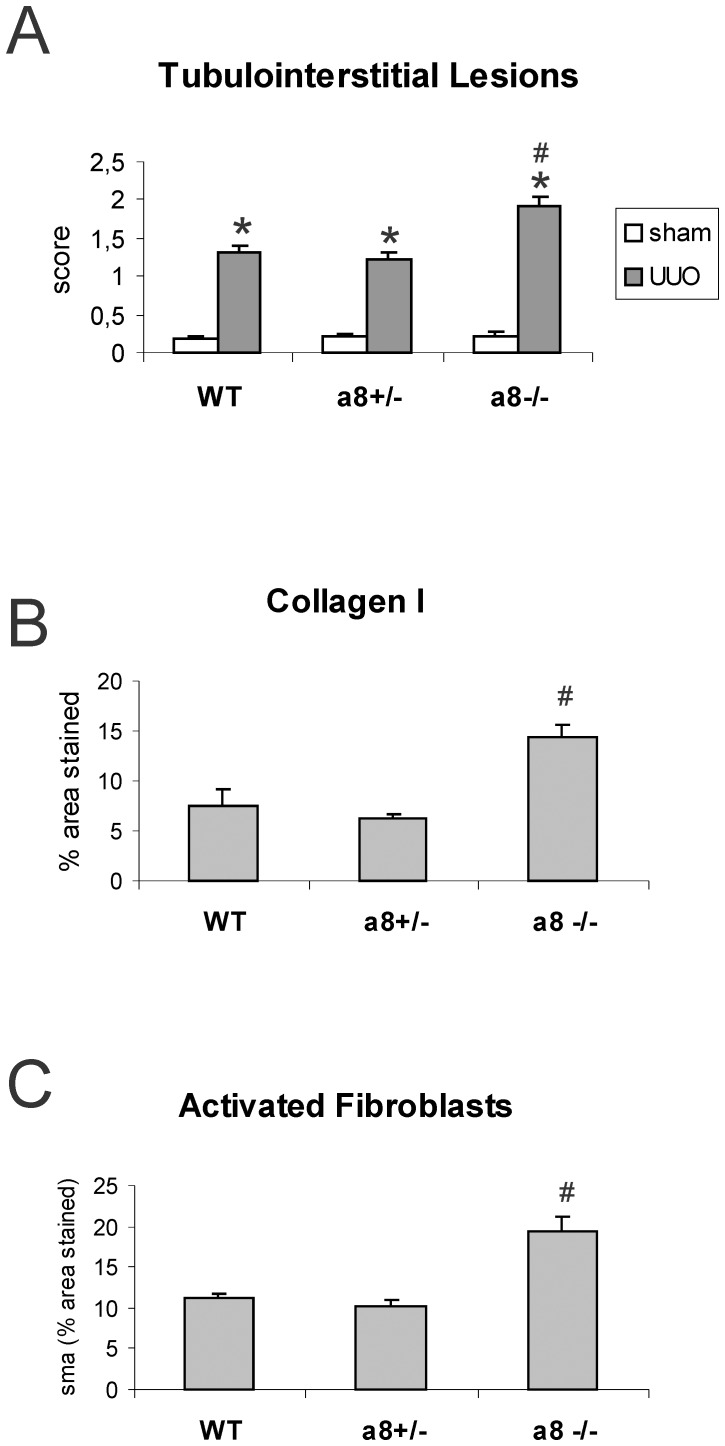
Tubulointerstitial changes in α8 integrin-deficient mice after unilateral ureteral obstruction (UUO). A: Tubulointerstitial lesion score. B: Expansion of tubulointerstitial collagen I deposition. C: Abundance of activated interstitial myofibroblasts as assessed after α-smooth muscle actin staining (sma). WT, wild type; α8+/−, mice with a heterozygous deficiency for α8 integrin; α8−/−, mice with a homozygous deficiency for α8 integrin. Data are means±sem. * p<0.05 UUO versus sham, # p<0.05 α8−/− versus wild type UUO.

**Figure 5:**
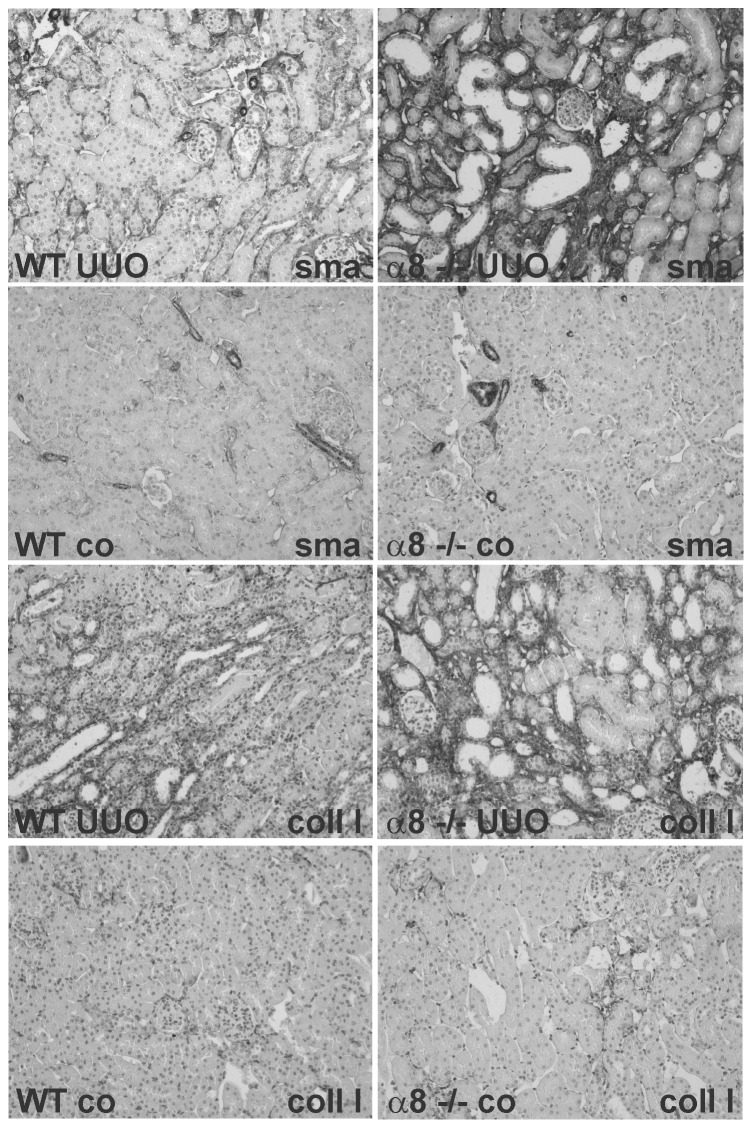
Representative examples of collagen I and α-smooth muscle actin stained renal sections of WT and α8 integrin-deficient mice. UUO, unilateral ureteral obstruction; co, control animal; WT, wild type; α8−/−, mice with a homozygous deficiency for α8 integrin; sma, α-smooth muscle actin; coll I, collagen I.

Tubulointerstitial damage as assessed by a tubulointerstitial lesion score was hardly detected and not different when control groups were compared, but increased significantly after UUO (figure 4A). No differences in tubulointerstitial damage were seen in wild type and α8+/− mice, but more prominent damage was detected in α8−/− mice compared to wild type or α8+/− mice after UUO (figure 4A). Consistent with this finding, tubulointerstitial collagen I expansion after UUO was the same in wild type and α8+/− mice, but more severe in α8−/− mice compared to wild type or α8+/− mice (figure 4B). Moreover, tubulointerstitial fibroblast activation to myofibroblasts was seen in all genotypes after UUO, but in α8−/− mice fibroblast activation was detected more frequently compared to wild type and α8+/− mice, which did not differ in the load of myofibroblasts (figure 4C). Exemplary photomicrographs showing the increase in collagen I and α-smooth muscle actin staining in kidney tissue of obstructed wild type and α8−/− mice are presented in figure 5. Supporting these findings, α-smooth muscle actin mRNA expression was significantly higher in obstructed α8−/− mice compared to wild types (table 3). Expression levels of collagen I mRNA, however, were induced in response to induction of UUO to a similar degree in all genotypes (table 3).

## Discussion

Our data show that α8 integrin was de novo expressed in tubular epithelial cells and interstitial fibroblasts in response to UUO. The α8 integrin ligands fibronectin, osteopontin and fibrillin-1 were upregulated and colocalized with α8 integrin in the tubulointerstitium. However, underexpression of α8 integrin did not attenuate renal fibrosis after UUO. On the other hand, the “extreme” genotype of mice with a homozygous deletion of α8 integrin, leading to the complete absence of the α8 integrin chain, induced more pronounced fibrotic changes in response to UUO. Thus, our results demonstrate that the α8 integrin chain is induced de novo in tubular epithelial cells and interstitial fibroblasts but this does not appear to add to interstitial fibrosis. Rather, the de novo expression of α8 integrin seems to play a protective role.

De novo expression of α8 integrin in tubulointerstitial fibrosis together with an increased expression of β1 integrin and α8β1 ligands, which also colocalize with α8 integrin to allow for α8 integrin-dependent signaling, seems to support the concept that α8 integrin may have a functional relevance for fibrotic alterations in the tubulointerstitium of the kidney. An increase in α8 integrin expression was detected in fibrotic events of other organs, like heart, liver and lung [14], [15] and was induced by profibrotic TGFβ and angiotensin II [13], [24]. Therefore, it was suggested to have profibrotic properties. The data of our present study, however, do not bear out this notion.

In accordance with our present data, hypertension-induced glomerulosclerosis was not attenuated in mice deficient for α8 integrin [25]. Overexpression of α8 integrin in the glomerulus, however, was exclusively due to an increased expression in mesangial cells which normally express α8 integrin, and did not involve de novo expression of this integrin in other glomerular cells [13]. This is in contrast to our findings in the tubulointerstitium where epithelial cells and fibroblasts, which are normaly negative for α8 integrin, de novo express this integrin. The α8 integrin chain is mainly found on mesenchymal cells [26], but some reports also exist describing epithelial α8 integrin expression [16], [27], [28] mainly in less differentiated epithelium. De novo expression of α8 integrin in tubular epithelial cells during fibrotic changes of the tubulointerstitium seems to support these previous findings. But a change in the degree of epithelial differentiation alone might not be sufficient to trigger an increase in fibrotic events.

Nevertheless, the lack of a clear profibrotic tubulointerstitial effect of α8 integrin signaling in our study was surprising in view of the many observations previously reported on related α integrin chains, the β1 integrin chain which dimerizes with the α8 integrin chain, and at least one ligand of α8 integrin in the UUO model: The α5 integrin chain which is closely related to α8 integrin is de novo expressed in tubular epithelial cells after UUO [5]. In UUO, tubulointerstitial expression of the β1 integrin chain increases and inhibition of β1 integrin signaling has antifibrotic effects [29]. The α8 integrin ligand osteopontin was also upregulated and colocalized with α8 integrin in tubular epithelial cells after UUO. Osteopontin was reported to contribute to tubulointerstitial fibrotic changes in the obstructed kidney [30]. Thus it seemed conceivable that these functions of osteopontin could occur via α8 integrin-dependent signaling. The data of our study, however, cast doubt on such an interrelation and argue for a signaling via other osteopontin receptors, like CD44 or αv integrins, to convey profibrotic effects.

A profibrotic function of α8 integrin in the development of tubulointerstitial fibrosis altogether seems unlikely, as in mice expressing reduced amounts of α8 integrin tubulointerstitial fibrosis is not attenuated. On the contrary, mice completely lacking α8 integrin developed a somewhat more severe fibrosis compared to mice with conserved renal expression of α8 integrin. Thus, α8 integrin could play a protective role in the tubulointerstitium. This might be due to an increased sensitivity of cells lacking α8 integrin for mechanical strain. In mice with a deficiency for α8 integrin more severe glomerular damage, i.e. disruption of the glomerular tuft, was detected in glomeruli after induction of glomerular hypertension [25] arguing for an important contribution of α8 integrin to the maintenance of tissue integrity. This might hold true not only for the glomerulus, but also for the tubulointerstitium, where α8 integrin is not regularly expressed. Similar protective functions were described for other integrin chains as well: Loss of α1 integrin results in more severe glomerulosclerosis after injury [7] and a selective deletion of the α3 integrin chain in podocytes also leads to increased accumulation of extracellular matrix in the glomerulus [31]. In this context one limitation has to be mentioned. We cannot completely rule out an effect of a somewhat reduced renal mass in α8 integrin-deficient mice. A reduced renal mass itself is known to aggravate the sequelae of renal injury, especially in the glomerulus [32]. It can further not be excluded that a more prominent role for α8 integrin exists in milder and more chronic forms of renal fibrosis, as UUO is a model of severe tubulointerstitial changes, which might override the effects of α8 integrin-mediated interactions. Finally, we cannot rule out that de novo expression of α8 integrin in tubular epithelial cells and activated interstitial fibroblasts is merely a marker of dedifferentiation in these cells not serving a special pathological function in tubulointerstitial fibrosis after UUO.

Taken together, our data do not support the hypothesis that de novo tubulointerstitial expression of α8 integrin contributes to fibrotic processes after tubulointerstitial injury. Thus, targeting α8 integrin does not seem to be a useful approach to attenuate renal fibrosis. More likely, the de novo expression of α8 integrin might serve protective functions as a complete loss of α8 integrin somewhat aggravates tubulointerstitial injury.
